# Decreased prefrontal activation during verbal fluency task after repetitive transcranial magnetic stimulation treatment for depression in Alzheimer’s disease: a functional near-infrared spectroscopy study

**DOI:** 10.3389/fnagi.2024.1460853

**Published:** 2025-01-08

**Authors:** Yuanzhi Zhao, Conglong Qiu, Ping Lin, Mei Yang, Ling Huang, Zheng Zhao, Xiangping Wu, Dongsheng Zhou

**Affiliations:** ^1^Department of Psychiatry, Affiliated Kangning Hospital of Ningbo University, Ningbo, China; ^2^Department of Psychiatry, Ningbo Kangning Hospital, Ningbo, China; ^3^Department of Psychiatry, Affiliated Women and Children's Hospital of Ningbo University, Ningbo, China

**Keywords:** depression in AD, repetitive transcranial magnetic stimulation, prefrontal cortex, functional near-infrared spectroscopy, verbal fluency task, bilateral standard rTMS

## Abstract

**Background:**

Studies have shown the clinical effects of repetitive transcranial magnetic stimulation (rTMS) on depression in Alzheimer’s disease (AD). However, the underlying mechanisms remain poorly understood. The measurement of brain activation links neurobiological and functional aspects but is challenging in patients with dementia. This study investigated the influence of rTMS on cortical activation in patients with AD and depressive symptoms, measured using functional near-infrared spectroscopy (fNIRS) during a verbal fluency task.

**Methods:**

In this randomized, double-blind study, patients with AD and depression received either active rTMS (n = 17) or sham-rTMS (n = 16). Patients received 4 weeks of bilateral standard rTMS (1 Hz rTMS delivered to the right dorsolateral prefrontal cortex (DLPFC) and 10-Hz rTMS delivered to the left DLPFC).

**Results:**

No significant changes were found in the Mini-Mental State Examination (MMSE) and Modified Barthel Index (MBI); however, significant changes were found for the 17-item Hamilton Depression Rating Scale (HAMD-17) and the depression score of the Neuropsychiatric Inventory (NPI-depression; *p* < 0.05). The results showed a decrease in the concentration of oxygenated hemoglobin, as measured with fNIRS, from baseline to week 4 in CH41 (in right DLPFC; *p* = 0.0047, FDR-corrected). There was a negative correlation between the improvement in HAMD-17 severity in these patients and reduced oxygenated hemodynamic response of CH41 (r = − 0.504, *p* = 0.039).

**Conclusion:**

The results indicated a positive effect of rTMS on depression in patients with AD. The underlying cortical changes were imaged using fNIRS. Prefrontal activation measured by fNIRS is a potential biomarker for monitoring the response of patients with depression in AD to rTMS treatment.

## 1 Introduction

Alzheimer’s disease (AD) is a primary neurodegenerative disease characterized by a progressive decline in short-term memory during its early stages. As AD progresses, cognitive function and daily social skills are further impaired (Botto et al., 2022). Behavioral and psychological symptoms in dementia (BPSD) have been suggested by the International Psychiatric Association to describe the spectrum of non-cognitive and non-neurological symptoms of dementia, such as agitation, aggression, mental illness, depression, and apathy (Padovani et al., 2023). Patients with AD have a higher incidence of BPSD than do older individuals without dementia (Lee and Lyketsos, 2003). Almost all patients with AD (97%) have BPSD (Takemoto et al., 2020). Depression is one of the most common forms of BPSD, affecting approximately 30–50% of patients (Teng et al., 2008). Typical depressive symptoms in patients with AD include insomnia, social withdrawal, reduced purpose-oriented behavior, loss of interest in once-enjoyable activities and hobbies, guilt, hopelessness, and sadness (Cimadamore et al., 2021). Depression not only exacerbates cognitive impairment in patients but also significantly impairs their daily living abilities, leading to a decrease in quality of life and increased risk of hospitalization and death. Conversely, treating the symptoms of depression that appear over the course of a neurodegenerative process can help delay the progression of dementia (Aguera-Ortiz et al., 2021).

Antidepressants are the main drugs used to treat depression in patients with AD. However, the efficacy of antidepressants for treating depression in patients with AD remains controversial (Orgeta et al., 2017). Additionally, older people have a higher risk than young people of adverse events when using antidepressants (Coupland et al., 2011). Therefore, comprehensive interventions, including noninvasive physical therapy, should be performed to treat depression in AD.

Transcranial magnetic stimulation (TMS) is a painless and non-invasive treatment that acts on the central nervous system, regulating the action potential of neurons, and affecting metabolism and neurophysiological activities in the brain. According to different TMS stimulation pulses, TMS can be divided into three stimulation modes: single-pulse TMS, paired-pulse TMS, and repetitive TMS (rTMS). rTMS is one of the most commonly used TMS approaches in clinical practice. Low frequency (≤1 Hz) stimulation can reduce cortical excitability, while high frequency (>1 Hz) stimulation can increase cortical excitability. Several studies have demonstrated therapeutic effects of rTMS on senile depression (Cappon et al., 2022). Additionally, rTMS not only improves cognition (Lin et al., 2019; Chou et al., 2020; Yan et al., 2023) but also prevents BPSD, especially depression, in patients with AD (Ahmed et al., 2012; Teselink et al., 2021; Zhang et al., 2022; Yang and Zhou, 2023).

Regional cerebral blood flow can serve as a biological marker to distinguish between patients with and without depression (Li et al., 2021), suggesting that functional near-infrared spectroscopy (fNIRS), a functional neuroimaging tool that investigates cerebral hemodynamic changes in the cerebral cortex, might be useful for evaluating the efficacy of rTMS for depression in patients (Gao et al., 2019; Xiong et al., 2023). For the underlying neuropathological association, the depression score was found to be correlated with brain regional tau deposition, especially in the temporal cortex including the entorhinal cortex and middle temporal cortex (Zhou, 2020).

In summary, in the current study, fNIRS was used as a detection technique, with cortical hemodynamics of the prefrontal and temporal cortices as clinical observation indicators, to explore whether the two cortices of patients with AD and depression have a specific response to rTMS treatment.

## 2 Materials and methods

### 2.1 Participants

This pilot feasibility study was a single-site, prospective, double-blind study in which patients and assessors were blinded, randomized, parallel-arm, and sham-controlled for rTMS treatment of depression in older adults with AD.

Patients were recruited from the Hospital Department of the Affiliated Kangning Hospital of Ningbo University from November 1, 2021, to November 4, 2022. Experienced research psychiatrists recommended that potential participants receive further study. Participants were randomized in a 1:1 ratio to either the active or sham-rTMS group using a computer-generated sequence. Allocation concealment was maintained by using sealed opaque envelopes. Participants received 20 consecutive 30-min applications of active/sham TMS from Monday to Friday for 4 weeks. A participant’s allocated intervention during the trial was revealed by the principal investigator at the end of the study. The trial results were communicated by the study coordinators when requested.

Inclusion criteria were as follows: (1) Participants who meet the criteria of probable AD defined by National Institute of Neurological and Communicative Diseases and Stroke-Alzheimer’s Disease and Related Disorders Association research criteria (McKhann et al., 1984); (2) Participants who met the criteria for depression in AD defined by the National Institute of Mental Health criteria (Teng et al., 2008); (3) Adequate visual and auditory abilities to perform all aspects of the cognitive and functional assessments, and sufficient mobility to allow transportation and participation in all planned interventions.

Exclusion criteria were as follows: (1) Life-threatening somatic diseases; (2) History of other mental disorders; (3) Alcohol or other substance abuse; (4) Disturbed consciousness, central nervous system infection, stroke, brain tumor, and other neurological diseases, or a history of diseases that may limit the use of rTMS or medical treatment devices, such as placement of cardiac pacemakers, intracranial metal, and aneurysm clips; and (5) Use of drugs or substances that affect cerebral perfusion, such as caffeine, alcohol, and acetazolamide within the day fNIRS was performed (Hernandez-Garcia et al., 2019). Prior to the study, all participants provided written informed consent. This study was approved by the Ethics Committee of the Hospital Department of the Affiliated Kangning Hospital of Ningbo University (Approval no.: NBKNYY-2021-LC-40) on November 1, 2021, and registered in the Chinese Clinical Trials Registry (registration no. ChiCTR2100053538) on November 24, 2021.

### 2.2 rTMS procedures (bilateral standard rTMS)

Participants received 20 consecutive 30-min applications of active/sham rTMS from Monday to Friday for 4 weeks (a total of 20 sessions). A figure-eight coil (Coil-D70-air film coil, Magstim) was placed over the left and right dorsolateral prefrontal cortex (DLPFC) lobes, which were determined using the MNI coordinates (MNIx,y,z = 44, 40, 29; Fox et al., 2012) in the neuronavigation Brainsight system (Rogue Research Inc., Montreal, Canada). Referring to the standard sequence of bilateral rTMS (Blumberger et al., 2022), the parameters of our research consisted of 1-Hz stimulation (120% resting motor threshold, 900 pulses over 15 min) to the right DLPFC, followed by standard FDA-cleared 10-Hz stimulation (120% resting motor threshold, 900 pulses over 15 min) to the left DLPFC. The sham treatments used a MAGSTIM pseudo-stimulus coil placed over the left and right DLPFC that transmitted no stimulation. The rTMS machine was a MAGSTIM Rapid2 model (Magstim Ltd., Oxford, UK). Patients in both groups experienced the same sound during the rTMS treatment. Participants were unaware of their assignation to sham or treatment group.

### 2.3 Neuropsychological assessment

All participants received neuropsychological and clinical evaluation, and data were collected via assessments that were implemented at baseline, and 2 weeks and 4 weeks after the end of treatment.

The primary outcome measure was the assessment of depression in AD. To reduce the error associated with using a single scale to evaluate depression in AD, this experiment used two scales: the Hamilton Depression Rating Scale (HAMD-17) and the Neuropsychiatric Inventory (NPI-depression; Cummings, 1997). A HAMD-17 score < 7 indicated no depression, with depression considered present for scores ≥7. The NPI is a proxy-reported scale developed to assess 12 neuropsychiatric disturbances that are common in dementia, of which depression is an important dimension.

Secondary outcome measures included the Mini-Mental State Examination (MMSE) and the Modified Barthel Index (MBI). The MMSE (Folstein et al., 1975) was used to assess general cognitive function. The MBI (Alsubiheen et al., 2022) was used to compare the level of ADL performance, with MBI scores ranging from 0 to 23.

Neuropsychological assessment was performed face-to-face by experienced psychiatrists blinded to the group allocation. During the interviews, demographic information, body weight, and height were measured and recorded. All assessors were trained at a monthly workshop.

### 2.4 Activation task (verbal fluency task)

**Figure 1:**
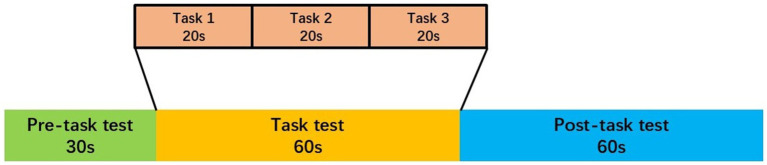
The VFT protocol used for near-infrared spectroscopy. Each trial consisted of a 30 s pre-task rest period, a 60 s task period subdivided into three 20 s task s and finally, a 60 s post-task rest period.

The task procedure used in the present study was a Chinese-language phonological verbal fluency task (VFT) developed by Quan et al. for Chinese participants (Quan et al., 2015). The VFT was executed during the daytime. The VFT consisted of a 30-s pre-task baseline, 60-s task period, and 60-s post-task baseline. During the pre- and post-task baseline periods, participants were asked to repeat counting from 1 to 5 following voice prompts from the fNIRS machine. During the task period, participants were required to construct as many phrases as possible using three commonly used characters, such as “蓝” (blue), “大” (big), and “天” (sky). Participants were instructed to generate as many words as possible, beginning with the same syllable. All participants were given the same syllable cues, and no changes were made to the order of presentation. We provided all participants with a practice session before formal testing to ensure that they fully understood the tasks. The three characters were changed every 20 s during the task period to reduce the time during which participants were silent (see Figure 1).

### 2.5 NIRS measurement

**Figure 2:**
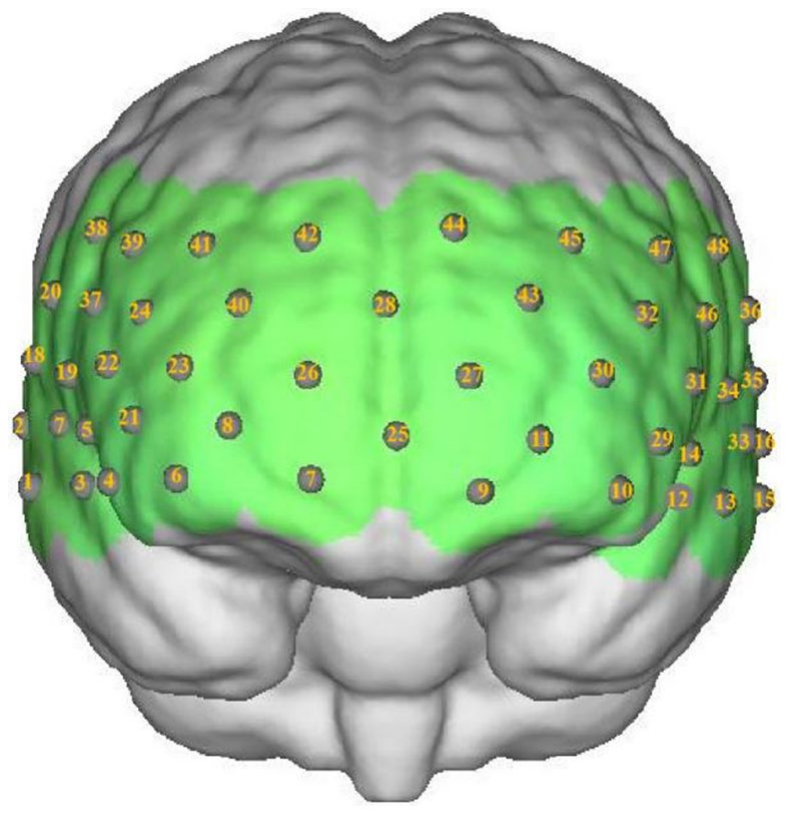
The position of channels.

Participants were seated comfortably in a quiet room. Hemoglobin concentrations were measured using a multichannel near-infrared optical imaging system (NirScan, Danyang Huichuang Medical Equipment Co., Ltd., China). The sampling frequency was 11 Hz, with major wavelengths of 730 and 850 nm, and 808 nm as the isotopic wavelength for correction. We used the FPz channel (10/20 International System) as the center of the middle probe; 31 SD probes (consisting of 15 sources and 16 detectors) with a fixed 3-cm inter-probe distance were placed to cover each participant’s bilateral PFC and temporal cortices, with the lowest probes positioned along the Fp1-Fp2 line (Figure 2). A total of 48 NIRS channels were established.

### 2.6 Data processing and analysis

#### 2.6.1 NIRS data analysis

The toolbox HOMER2, a MATLAB-based graphical user interface program, was used to analyze NIRS data (Huppert et al., 2009). Data were preprocessed using the following steps: motion artifacts were corrected using moving SD and cubic spline interpolation methods. A 0.01–0.20 Hz bandpass filter was used to remove physiological noise (e.g., respiration, cardiac activity, and low-frequency signal drift). The modified Beer–Lambert law was used to convert optical densities into changes in oxygenated hemoglobin (oxy-Hb) and deoxygenated hemoglobin (deoxy-Hb) concentrations. We used oxy-Hb as our primary indicator in the following analysis because the change in oxy-Hb could better reflect cortical activity, as it is assumed to more directly respond to cognitive task-related brain activation and more strongly correlate with blood oxygenation level-dependent signals measured by fMRI (Strangman et al., 2002). We used the final 10 s of the pre-task rest period as the baseline. The VFT block waveforms were calculated using a block range set of 0–125 s, pre-baseline range set of 0–10 s, and post-baseline range set of 70–125 s. We used a 60-s task period to construct the time window to analyze the mean oxy-Hb changes. Linear fitting was applied to the data of the two baselines. According to the waveforms of individuals in all 48 channels, the average waveforms of oxy-Hb and deoxy-Hb changes in all participants in the two groups were obtained.

#### 2.6.2 Statistics

Statistical analyses were conducted using SPSS 22.0 (IBM Corp., NY, USA). The NirSpark software package and GraphPad Prism 8 were used to generate figures and graphs, respectively. Data normality was tested using the Shapiro–Wilk test. The demographic and clinical data were analyzed using a chi-squared test, t-test, or Mann–Whitney U test to compare the rTMS and sham-rTMS groups. We used a two-way mixed ANOVA with different groups (rTMS group vs. sham-rTMS group) as the between-participants factor and time (pre vs. post) as the within-participants factor to analyze the effect of rTMS intervention on neuropsychological assessment. The sphericity of the set of variables was evaluated using the Mauchly test, and, when it was violated, the Greenhousee-Geisser correction was used. The effect size of the mixed-design ANOVA was determined using partial eta squared (η^2^). Pairwise multiple comparisons between follow-up time points and baseline were conducted within each group and adjusted with Bonferroni procedure. To analyze our fNIRS data, independent samples t-tests were used to compare oxy-Hb values during the VFT for each channel between the rTMS and sham-rTMS at baseline and 4 weeks. The differences in oxy-Hb values during the VFT for each channel were compared between pre- and post-treatment, using paired t-tests. In case of non-normal data, non-parametric Mann Whitney U test and Wilcoxon signed-rank test were used as appropriate. Cohen’s d effect size was used to measure the magnitude of the difference between groups. The statistical results were corrected for multiple comparision across channels by using the false discovery rate (FDR) controlling procedure. Pearson’s correlation coefficient was performed to determine the relationship between oxy-Hb change values (post-pre) and HAMD and NPI-depression change scores (post-pre). Statistical significance was defined as *p* < 0.05, two-tailed.

## 3 Results

**Figure 3:**
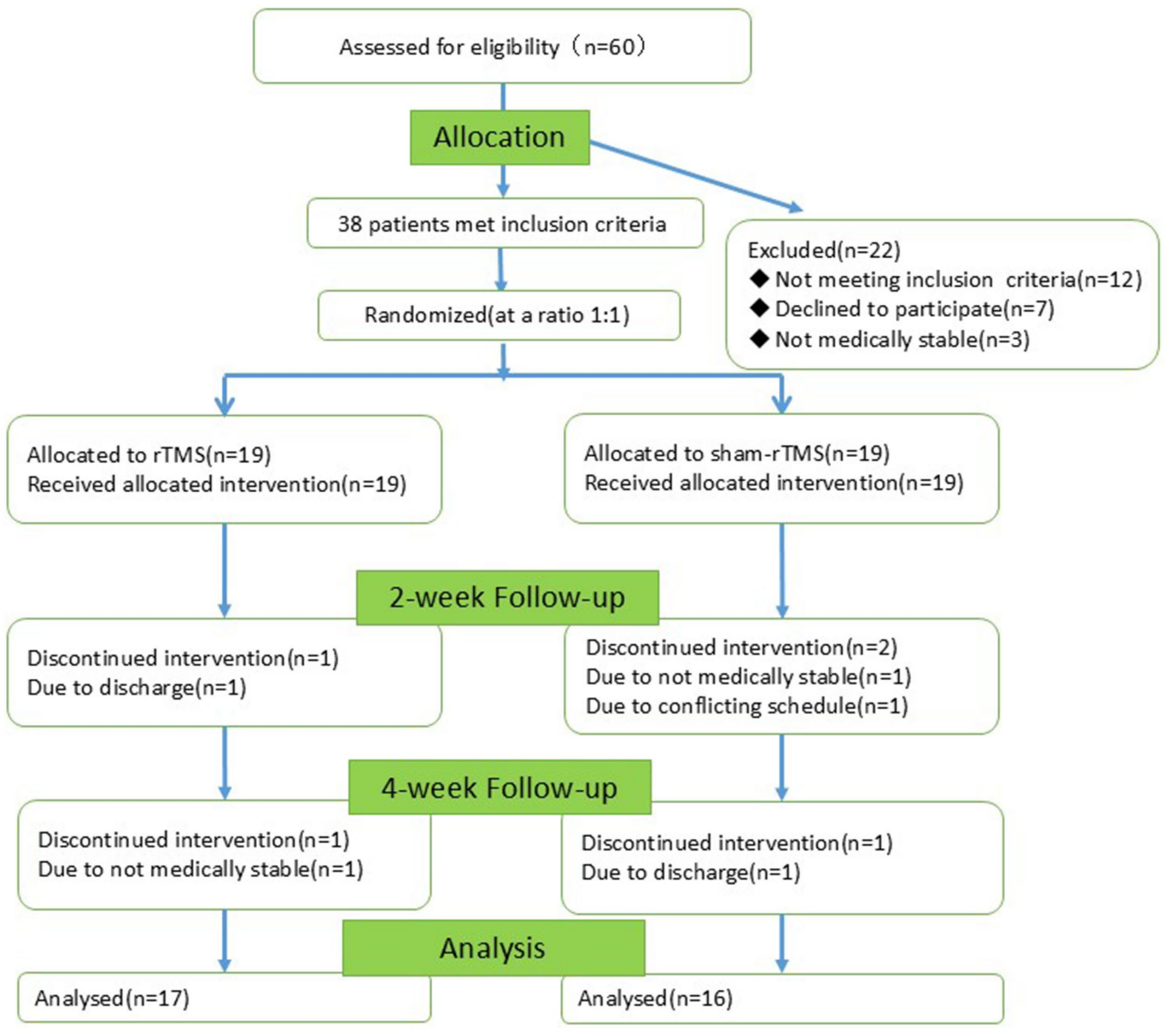
CONSORT flow diagram of participants through the trial.

In total, 60 patients with AD were recruited, of whom 22 were excluded (12 did not meet the inclusion criteria, 7 declined to participate and 3 had unstable medical conditions). The remaining 38 individuals were randomly divided into two groups (19 in the rTMS group and 19 in the sham-rTMS group). During the experiment, five individuals were lost (two from the rTMS group and three from the sham-rTMS group). Screening, enrollment, and participation are shown in Figure 3. The final 33 patients completed the 2-week intervention and the 4-week follow-up.

### 3.1 Demographic and clinical characteristics

Patient baseline characteristics are detailed in Table 1. Mean patient age was 73.76 ± 3.882 and 72.63 ± 6.752 years old in the rTMS Group and Sham-rTMS Group, respectively. There were 7/10 (41.2%) and 7/9 (43.8%) males/females in the rTMS Group and Sham-rTMS Group, respectively. There were no significant differences between the two groups in terms of demographics, concomitant medications, or comorbidities.

**Table 1:** Baseline patients’ demographic and clinical characteristics.

Variables	rTMS Group (*n* = 17)	Sham-rTMS Group (*n* = 16)	*X*^2^/z/t	*p*-value
Age, years, mean (SD)	73.76 (3.882)	72.63 (6.752)	1.140	0.267
Gender, male/female	7/10	7/9	0.036	0.849
BMI, kg/m^2^, mean (SD)	23.565 (4.5958)	21.737 (2.4309)	1.439	0.163
Education, years, median (interquartile range, IQR)	9 (6)	9 (3)	−0.379	0.705
Years since diagnosis of Alzheimer’s disease, median (IQR)	2.0 (2.0)	2.0 (3.0)	−0.391	0.695
Concomitant medications				
Acetylcholinesterase Inhibitors, *n* (%)	13 (76.5%)	12 (75%)	0.010	0.922
Memantine, *n* (%)	12 (70.6%)	11 (68.8%)	0.013	0.909
Antidepressants, *n* (%)	11 (64.7%)	11 (68.8%)	0.061	0.805
Complications (diabetes, hypertension, or both), *n* (%)	10 (58.8%)	11 (68.8%)	0.351	0.554

### 3.2 Clinical outcomes

#### 3.2.1 Primary outcomes

**Figure 4:**
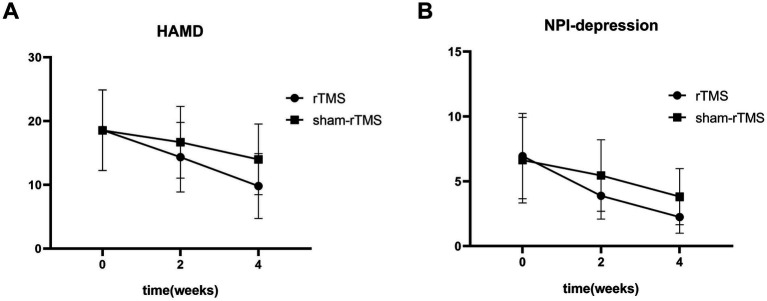
The HAMD score **(A)** and NPI-depression score **(B)** of patients at baseline, and weeks 2 and 4 after treatment.

Figure 4 shows the primary results. Tables 2, 3 shows the two-way repeated-measures ANOVA results for the HAMD and NPI-depression scores in the two groups.

**Table 2:** Primary outcomes.

Scales	rTMS Group (*n* = 17)			Sham-rTMS Group (*n* = 16)			Time	Group	Time × Group
	Baseline	2 weeks	4 weeks	Baseline	2 weeks	4 weeks	P, F, η^2^	P, F, η^2^	P, F, η^2^
HAMD, mean (SD)	18.59 (6.315)	14.35 (5.454)	9.82 (5.09)	18.56 (6.314)	16.69 (5.618)	14 (5.538)	<0.01, 141.126, 0.823	0.275, 1.236, 0.038	<0.01, 14.07, 0.312
NPI-depression, mean (SD)	6.94 (3.288)	3.88 (1.799)	2.24 (1.251)	6.63 (3.304)	5.44 (2.756)	3.81 (2.167)	<0.01, 61.046, 0.803	0.264, 1.297, 0.04	0.004, 7.784, 0.202

**Table 3:** Pairwise multiple comparison results related to the primary outcomes.

Scales	Groups	Times, mean (SD)			2 weeks vs. Baseline			4 weeks vs. Baseline		
		Baseline	2 weeks	4 weeks	Mean (2 weeks − baseline)	95% CI	*p*	Mean (4 weeks − baseline)	95% CI	*p*
HAMD	TMS Group	18.588 (1.531)	14.353 (1.342)	9.824 (1.288)	−4.235	−5.342 to −3.128	<0.001	−8.765	−10.639 to −6.890	<0.001
	Sham-rTMS Group	18.563 (1.579)	16.688 (1.383)	14.000 (1.328)	−1.875	−3.016 to −0.734	0.001	−4.563	−6.494 to −2.631	<0.001
NPI-depression	TMS Group	6.941 (0.799)	3.882 (0.561)	2.235 (0.426)	−3.059	−4.016 to −2.102	<0.001	−4.706	−5.933 to −3.478	<0.001
	Sham-rTMS Group	6.625 (0.824)	5.438 (0.578)	3.813 (0.439)	−1.188	−2.174 to −0.201	0.014	−2.813	−4.078 to −1.547	<0.001

All the p-values are adjusted with Bonferroni procedure.

Repeated measures analysis revealed a significant time × group interaction (*F* = 14.07, *p* < 0.01) and an effect of time (*F* = 141.12, *p* < 0.01) on HAMD scores in patients. However, there was no significant difference in HAMD scores between the two groups (*F* = 1.236, *p* = 0.275). Simple effects analyses revealed that the HAMD scores in the rTMS group were significantly lower than those in the sham-rTMS group at week 4 (*p* = 0.031). Multiple comparisons showed that the HAMD scores in the rTMS and sham-rTMS groups were significantly reduced at weeks 2 and 4, respectively, compared to baseline.

Repeated measures analysis revealed a significant time × group interaction (*F* = 7.784, *p* = 0.004) and an effect of time (*F* = 61.046, *p* < 0.01) on NPI-depression scores in patients. Similarly, there was no significant difference in NPI-depression scores between the two groups (*F* = 1.297, *p* = 0.264). Additionally, NPI-depression scores in the rTMS group were significantly lower than those in the sham-rTMS group at week 4 (*p* = 0.015). The NPI-depression scores in the rTMS and sham-rTMS groups were significantly reduced at weeks 2 and 4, respectively, compared with baseline.

#### 3.2.2 Secondary outcomes

The secondary outcomes are presented in Table 4. Repeated-measures analysis revealed that no effects were significant (time × group, *F* = 0.400, *p* = 0.599; time, *F* = 2.653, *p* = 0.099; group, *F* = 0.233, *p* = 0.633) on MMSE scores in patients. Repeated measures analysis revealed a significant effect of time (*F* = 6.610, *p* = 0.006) on MBI scores in patients. Multiple comparisons showed that the MBI scores in the TMS and sham-TMS groups were significantly reduced at weeks 4 compared to baseline (*p* = 0.019). The time by group interaction (*F* = 0.549, *p* = 0.533) and the difference between the two groups (*F* = 0.260, *p* = 0.614) were not significant.

**Table 4:** Secondary outcomes.

Scales	rTMS Group (*n* = 17)			Sham-rTMS Group (*n* = 16)			Time	Group	Tim × Group
	Baseline	2 weeks	4 weeks	Baseline	2 weeks	4 weeks	P, F, η^2^	P, F, η^2^	P, F, η^2^
MMSE, mean (SD)	17.65 (4.197)	17.88 (4.196)	17.94 (4.451)	17.06 (3.043)	17.31 (3.092)	17.19 (3.487)	0.099, 2.653, 0.079	0.633, 0.233, 0.007	0.599, 0.400, 0.013
MBI, mean (SD)	82.35 (13.477)	82.94 (12.382)	84.12 (11.757)	85.00 (17.795)	85.94 (16.147)	86.25 (15.759)	0.006, 6.610, 0.176	0.614, 0.260, 0.008	0.533, 0.549, 0.017

### 3.3 Effects of rTMS on oxy-Hb signals during VFT task

**Figure 5:**
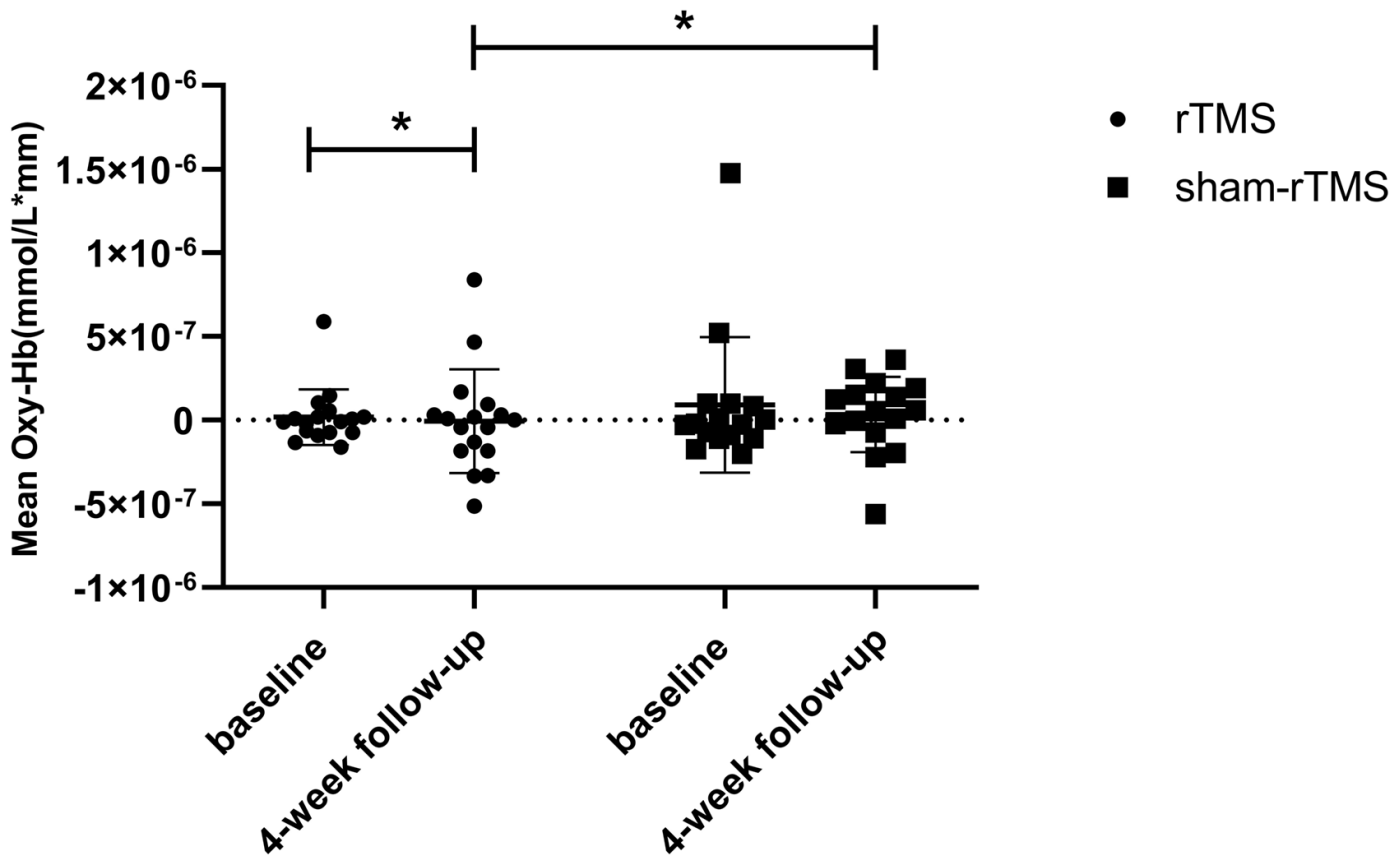
Comparing the difference of concentration of HbO in rTMS group and the sham-rTMS group in channel 41. The statistical threshold was set at *p* < 0.05. *p < 0.05.

The mean baseline oxy-Hb concentrations for CH 41 were not significantly different between the rTMS and sham-rTMS groups (*p* = 0.496, FDR-corrected). We found a lower mean oxy-Hb signal in the rTMS group than in the sham-rTMS group at week 4 (*p* = 0.0155, FDR-corrected; Cohen’s d = 0.228; Figure 5). The rTMS group showed a significant difference after 4 weeks of treatment (*p* = 0.0047, FDR-corrected), whereas no changes were found in the sham-rTMS group after 4 weeks of treatment (*p* = 0.583, FDR-corrected; Cohen’s d = 0.230). We simultaneously analyzed the other 47 channels and found no significant differences in the concentrations of oxy-Hb between the rTMS and sham-rTMS groups.

### 3.4 Correlation between primary outcomes change and oxy-Hb change

**Figure 6:**
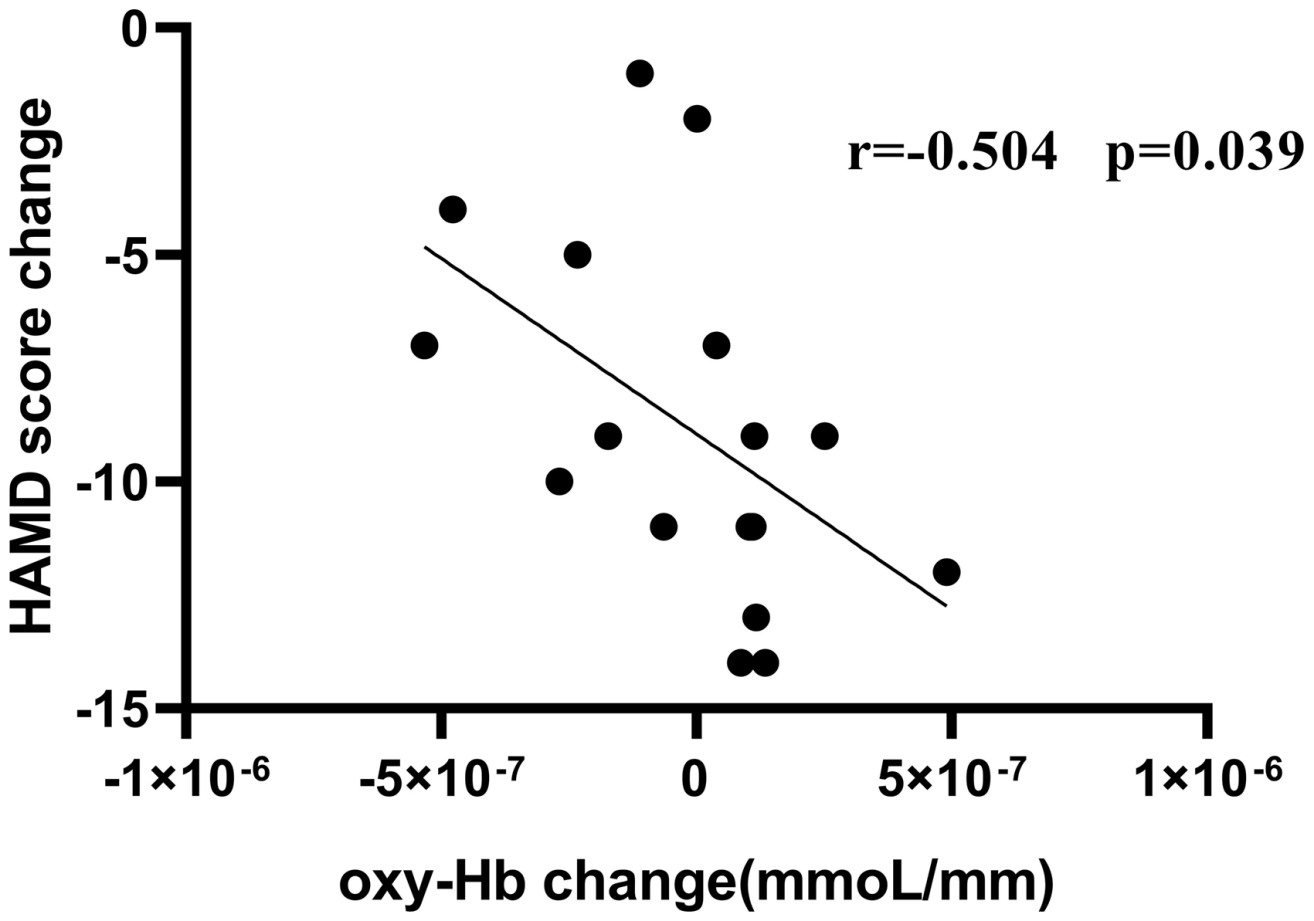
Correlation Between HAMD score change and oxy-Hb change.

Figure 6 shows that there was a negative correlation between the improvement in HAMD severity in these patients and reduced oxy-Hb concentrations of CH41 (r = −0.504, *p* = 0.039). A non-significan correlation between the improvement in NPI-depression severity and reduced oxy-Hb concentrations was observed (r = −0.426, *p* = 0.0878).

### 3.5 Adverse events

The procedure was safe and well-tolerated. Five participants reported adverse events, four in the rTMS group and one in the sham-rTMS group. All events were mild and mostly resolved on the day of occurrence with either minor or no action (mild headache, *n* = 3; scalp/skin discomfort, *n* = 3; neck pain/stiffness, *n* = 1; fatigue, *n* = 1). Details of adverse events are listed in Table 5.

**Table 5:** Adverse events by relationship to study device and study group.

Adverse events	rTMS Group (*n* = 17)	Sham-rTMS Group (*n* = 16)
Headache	2	1
Scalp/skin discomfort	2	1
Neck pain/stiffness	1	0
Fatigue	0	1

## 4 Discussion

This study found that patients with depression in AD who received rTMS treatment showed significant improvement in depression compared to the sham-TMS group. A small number of RCT have been performed of rTMS applied to patients with depression in AD. Ahmed et al. showed that high-frequency rTMS applied bilaterally to the DLPFC improved Geriatric Depression Scale scores in patients with AD (Ahmed et al., 2012). Lee et al. found that the Geriatric Depression Scale score did not improve significantly in a rTMS-COG (rTMS combined with cognitive training) treatment group (Lee et al., 2016). The main reason for this is likely that the treatment parameters (especially brain regions stimulated) of rTMS differ significantly from those in our trial.

Many studies have shown that the PFC affects individuals’ emotions and behaviors, and a significant relationship exists between abnormal PFC function and cognitive defects in patients with depression (Akiyama et al., 2018; Gao et al., 2022). For instance, significant connection between dorso-lateral prefrontal cortex (DLPFC) and depression score has been reported in a general clinical population including multiple sclerosis (MS) patients (Zhou, 2019). Some studies have found that significantly reduced frontotemporal activation, including the left DLPFC, is the key to the onset of depression (Akiyama et al., 2018; Tsujii et al., 2021). Others have indicated that the right PFC plays a key role in the development of depression (Cao et al., 2013; Tsujii et al., 2021). It is generally believed that high-frequency TMS of the left DLPFC or low-frequency TMS of the right DLPFC can be used to treat depression (Cao et al., 2013).

Currently, most studies have used rTMS to stimulate the left DLPFC to treat AD; however, there are relatively few reports on its therapeutic effects on depression in AD (Xue et al., 2024). Thus, a single stimulus target may not be effective for treating depression in patients with AD. Standard bilateral rTMS (high-frequency rTMS stimulation of the left DLPFC combined with low-frequency rTMS stimulation of the right DLPFC) has been shown to be efficacious in multiple clinical trials, and is one of the most effective rTMS protocols according to network meta-analyses (Mutz et al., 2019). One study found superior remission rates with bilateral stimulation (40%) compared with both left-unilateral (0%) and sham (0%) stimulation in older patients with TRD (Trevizol et al., 2019). Ahmed et al. found that high-frequency stimulation of the left and right DLPFC improved cognition and depression in patients with AD (Ahmed et al., 2012). Although that study suggests that bilateral DLPFC stimulation can improve depression in patients with AD, the patients were not treated with standard bilateral rTMS. Therefore, the current study is the first to use standard bilateral rTMS to treat depression in AD and to explore its possible cortical activation mechanism.

Our research found that the cognitive ability of patients with AD who received MMSE assessment showed little or no significant improvement after 4 weeks of rTMS treatment compared with the sham group, which is consistent with the results of a meta-analysis (Dong et al., 2018). The main reasons include the following: the MMSE is relatively less sensitive for cognitive assessment of AD, and some subtle cognitive improvements cannot be detected. The ADAS-Cog score and other scales are more precise than is the MMSE for exploring cognitive function (Dong et al., 2018). The duration of rTMS intervention was only 4 weeks; if it reaches 6 weeks or more, the improvement may be statistically significant. The purpose of this intervention was more inclined toward improving depression, and there are differences in the parameters of the TMS intervention in this experiment. Patients with severe dementia are not suitable for rTMS treatment (Sabbagh et al., 2020). Some patients in this trial had severe dementia, which may have affected the rTMS treatment outcomes.

No significant improvement in MBI was observed among patients with AD in this study after treatment, and the differences between the treatment and sham groups were not significant. This is consistent with the results of several studies (Dong et al., 2018). The most likely reason for this is that the MBI is not well-adapted to the assessment of ADL in patients with AD.

Studies have found a significant relationship between changes in hemodynamics in the right DLPFC and the severity of depressive symptoms (Noda et al., 2012). Arai et al. found lower activation of the bilateral frontal and parietal lobes in patients with AD during a VFT (Arai et al., 2006). Yap et al. observed lower and relatively delayed activation of the left PFC during a VFT in patients with AD (Yap et al., 2017). Metzger et al. showed hypoactivation of frontoparietal areas (such as the DLPFC) during the VFT in AD (Metzger et al., 2016). Herrmann et al. found reduced DLPFC and less locally specific activation during the VFT in patients with AD (Yap et al., 2017).

Compared to more well-known technologies, such as magnetic resonance imaging and positron emission tomography, fNIRS has multiple practical advantages: it is noninvasive, easy to use, low-cost, and portable. Another important advantage of fNIRS is its relatively low sensitivity to motion, which permits the adoption of more ecologically effective tasks. This is particularly important for patients with dementia who cooperate poorly with data collection. Therefore, NIRS has great potential for the diagnosis and evaluation of neurocognitive and motor dysfunctions (Pinti et al., 2020).

Many of the aforementioned studies combined the VFT paradigm with fNIRS technology and found that the activation patterns in the left DLPFC are closely related to both AD and depression (Yap et al., 2017; Akiyama et al., 2018; Tsujii et al., 2021), whereas the activation patterns in the right DLPFC are closely related to depression (Noda et al., 2012).

Interestingly, this study’s results showed that, compared with the sham-TMS group, the average hemoglobin concentration of channel 41 in the TMS group decreased significantly after 4 weeks of treatment; this channel was located in the right DLPFC (Li et al., 2024). The decrease in channel 41 activation from baseline to post treatment negatively correlated with the improvement in depressive symptoms. Compared with other studies, this study did not detect activation of the DLPFC in either hemisphere (Burke et al., 2022; Huang et al., 2022).The right DLPFC itself is related to negative emotions such as depression. Low-frequency TMS can improve depression by reducing cerebral blood flow in the right DLPFC and other brain areas (Kito et al., 2008; Kito, 2012).

The relative maintenance of cognitive function, along with greater hemodynamic responses (hyperactivation) following fNIRS, suggests the involvement of compensatory mechanisms (Clement and Belleville, 2010). However, failure of neural compensation (reduced hemodynamic responses and hypoactivation) is predominantly observed in the more severe stages of neurodegeneration (Niu et al., 2013). Patients with AD are prone to complete interruption of compensatory responses due to their inability to cope with excessive cognitive load and may have difficulty activating brain function during a VFT. Short-term, low-intensity rTMS combined with drug stimulation may cause difficulty in achieving statistically significant cognitive improvement and increased left DLPFC activation.

Our study provides the first evidence of a correlation between reduced fNIRS activation in the specific right DLPFC region and improvement in depressive symptoms in patients during rTMS treatment. fNIRS can be used to monitor the therapeutic response of rTMS treatment in patients with AD and depression. Our observations support the potential mechanism by which rTMS improves depression in AD, which is to reduce metabolic activity and blood flow perfusion in specific regions of the right DLPFC.

### 4.1 Limitations

The current study has some limitations. Our sample size was small, and future studies with larger sample sizes are required to confirm these preliminary findings. We did not evaluate behavioral performance on the VFT. Some patients with AD have difficulty completing the VFT owing to poor cognition, which could affect the NIRS assessment results. Additional paradigms may be required in the future to improve the results’ accuracy and reliability. Participants were all hospitalized patients and, therefore, it would have been difficult to require patients to return to the hospital for follow-up after discharge. Hence, the patients were not further followed up after the end of the study; hence, we cannot know the long-term effects of the treatment in this trial, and given the progressive course of AD, it is likely that symptoms worsened again once the interventions were stopped. Having more frequent fNIRS measurements (e.g., weekly) in longitudinal studies may permit better understanding of brain dynamics and minimize the influence of confounding factors.

## 5 Conclusion

In summary, an effect of TMS was observed in a small sample of patients with AD. Using fNIRS technology, we found that patients with depression in AD had significantly reduced right DLPFC-specific brain activation during the VFT period after bilateral standard rTMS. There was a correlation between the improvement in depression severity in these patients and the reduced oxy-Hb rresponse of specific brain regions in the right DLPFC. These results indicate that using fNIRS to measure the hemodynamic response in the PFC to a VFT is a potential biomarker for monitoring patients’ response to rTMS. Improving cognition, depression, and brain function in AD and predicting patient prognosis are important issues that require further exploration.

## Data Availability

The original contributions presented in the study are included in the article/supplementary material, further inquiries can be directed to the corresponding authors.

## References

[ref1] Aguera-Ortiz L. Garcia-Ramos R. Grandas Perez F. J. Lopez-Alvarez J. Montes Rodriguez J. M. Olazaran Rodriguez F. J.. (2021). Depression in Alzheimer's disease: a Delphi consensus on etiology, risk factors, and clinical management. Front. Psychol. 12:638651. doi: 10.3389/fpsyt.2021.638651, PMID: 33716830 PMC7953133

[ref2] Ahmed M. A. Darwish E. S. Khedr E. M. El Serogy Y. M. Ali A. M. (2012). Effects of low versus high frequencies of repetitive transcranial magnetic stimulation on cognitive function and cortical excitability in Alzheimer's dementia. J. Neurol. 259, 83–92. doi: 10.1007/s00415-011-6128-4, PMID: 21671144

[ref3] Akiyama T. Koeda M. Okubo Y. Kimura M. (2018). Hypofunction of left dorsolateral prefrontal cortex in depression during verbal fluency task: a multi-channel near-infrared spectroscopy study. J. Affect. Disord. 231, 83–90. doi: 10.1016/j.jad.2018.01.010, PMID: 29455100

[ref4] Alsubiheen A. M. Choi W. Yu W. Lee H. (2022). The effect of task-oriented activities training on upper-limb function, daily activities, and quality of life in chronic stroke patients: a randomized controlled trial. Int. J. Environ. Res. Public Health 19:4125. doi: 10.3390/ijerph192114125, PMID: 36361001 PMC9654844

[ref5] Arai H. Takano M. Miyakawa K. Ota T. Takahashi T. Asaka H.. (2006). A quantitative near-infrared spectroscopy study: a decrease in cerebral hemoglobin oxygenation in Alzheimer's disease and mild cognitive impairment. Brain Cogn. 61, 189–194. doi: 10.1016/j.bandc.2005.12.012, PMID: 16466836

[ref6] Blumberger D. M. Mulsant B. H. Thorpe K. E. Mcclintock S. M. Konstantinou G. N. Lee H. H.. (2022). Effectiveness of standard sequential bilateral repetitive transcranial magnetic stimulation vs bilateral Theta burst stimulation in older adults with depression: the FOUR-D randomized noninferiority clinical trial. JAMA Psychiatry 79, 1065–1073. doi: 10.1001/jamapsychiatry.2022.2862, PMID: 36129719 PMC9494264

[ref7] Botto R. Callai N. Cermelli A. Causarano L. Rainero I. (2022). Anxiety and depression in Alzheimer's disease: a systematic review of pathogenetic mechanisms and relation to cognitive decline. Neurol. Sci. 43, 4107–4124. doi: 10.1007/s10072-022-06068-x, PMID: 35461471 PMC9213384

[ref8] Burke M. J. Romanella S. M. Mencarelli L. Greben R. Fox M. D. Kaptchuk T. J.. (2022). Placebo effects and neuromodulation for depression: a meta-analysis and evaluation of shared mechanisms. Mol. Psychiatry 27, 1658–1666. doi: 10.1038/s41380-021-01397-3, PMID: 34903861

[ref9] Cao T. T. Thomson R. H. Bailey N. W. Rogasch N. C. Segrave R. A. Maller J. J.. (2013). A near infra-red study of blood oxygenation changes resulting from high and low frequency repetitive transcranial magnetic stimulation. Brain Stimul. 6, 922–924. doi: 10.1016/j.brs.2013.04.006, PMID: 23721908

[ref10] Cappon D. Den Boer T. Jordan C. Yu W. Metzger E. Pascual-Leone A. (2022). Transcranial magnetic stimulation (TMS) for geriatric depression. Ageing Res. Rev. 74:101531. doi: 10.1016/j.arr.2021.101531, PMID: 34839043 PMC8996329

[ref11] Chou Y. H. Ton That V. Sundman M. (2020). A systematic review and meta-analysis of rTMS effects on cognitive enhancement in mild cognitive impairment and Alzheimer's disease. Neurobiol. Aging 86, 1–10. doi: 10.1016/j.neurobiolaging.2019.08.020, PMID: 31783330 PMC6995441

[ref12] Cimadamore A. Lopez-Beltran A. Scarpelli M. Montironi R. (2021). RE: noninvasive papillary urothelial neoplasia (NIPUN): renaming cancer, by Jones TD and Cheng L, https://doi.org/10.1016/j.urolonc.2020.12.007 (low grade papillary intra-urothelial neoplasia). Urol. Oncol. 39, 308–309. doi: 10.1016/j.urolonc.2021.02.010, PMID: 33674150

[ref13] Clement F. Belleville S. (2010). Compensation and disease severity on the memory-related activations in mild cognitive impairment. Biol. Psychiatry 68, 894–902. doi: 10.1016/j.biopsych.2010.02.004, PMID: 20359695

[ref14] Coupland C. Dhiman P. Morriss R. Arthur A. Barton G. Hippisley-Cox J. (2011). Antidepressant use and risk of adverse outcomes in older people: population based cohort study. BMJ 343:d4551. doi: 10.1136/bmj.d4551, PMID: 21810886 PMC3149102

[ref15] Cummings J. L. (1997). The neuropsychiatric inventory: assessing psychopathology in dementia patients. Neurology 48, S10–S16. doi: 10.1212/WNL.48.5_Suppl_6.10S, PMID: 9153155

[ref16] Dong X. Yan L. Huang L. Guan X. Dong C. Tao H.. (2018). Repetitive transcranial magnetic stimulation for the treatment of Alzheimer's disease: a systematic review and meta-analysis of randomized controlled trials. PLoS One 13:e0205704. doi: 10.1371/journal.pone.0205704, PMID: 30312319 PMC6185837

[ref17] Folstein M. F. Folstein S. E. Mchugh P. R. (1975). "Mini-mental state". A practical method for grading the cognitive state of patients for the clinician. J. Psychiatr. Res. 12, 189–198. doi: 10.1016/0022-3956(75)90026-6, PMID: 1202204

[ref18] Fox M. D. Buckner R. L. White M. P. Greicius M. D. Pascual-Leone A. (2012). Efficacy of transcranial magnetic stimulation targets for depression is related to intrinsic functional connectivity with the subgenual cingulate. Biol. Psychiatry 72, 595–603. doi: 10.1016/j.biopsych.2012.04.028, PMID: 22658708 PMC4120275

[ref19] Gao L. Cai Y. Wang H. Wang G. Zhang Q. Yan X. (2019). Probing prefrontal cortex hemodynamic alterations during facial emotion recognition for major depression disorder through functional near-infrared spectroscopy. J. Neural Eng. 16:026026. doi: 10.1088/1741-2552/ab0093, PMID: 30669122

[ref20] Gao C. Zhou H. Liu J. Xiu J. Huang Q. Liang Y.. (2022). Characteristics of frontal activity relevant to cognitive function in bipolar depression: an fNIRS study. Biomed. Opt. Express 13, 1551–1563. doi: 10.1364/BOE.448244, PMID: 35414983 PMC8973170

[ref21] Hernandez-Garcia L. Lahiri A. Schollenberger J. (2019). Recent progress in ASL. NeuroImage 187, 3–16. doi: 10.1016/j.neuroimage.2017.12.095, PMID: 29305164 PMC6030511

[ref22] Huang J. Zhang J. Zhang T. Wang P. Zheng Z. (2022). Increased prefrontal activation during verbal fluency task after repetitive transcranial magnetic stimulation treatment in depression: a functional near-infrared spectroscopy study. Front. Psychol. 13:876136. doi: 10.3389/fpsyt.2022.876136, PMID: 35444573 PMC9013767

[ref23] Huppert T. J. Diamond S. G. Franceschini M. A. Boas D. A. (2009). HomER: a review of time-series analysis methods for near-infrared spectroscopy of the brain. Appl. Opt. 48, D280–D298. doi: 10.1364/AO.48.00D280, PMID: 19340120 PMC2761652

[ref24] Kito S. (2012). Treatment of depression using transcranial stimulation (TMS) and neuroimaging. Seishin Shinkeigaku Zasshi 114, 601–607, PMID: 22950163

[ref25] Kito S. Fujita K. Koga Y. (2008). Regional cerebral blood flow changes after low-frequency transcranial magnetic stimulation of the right dorsolateral prefrontal cortex in treatment-resistant depression. Neuropsychobiology 58, 29–36. doi: 10.1159/000154477, PMID: 18781088

[ref26] Lee J. Choi B. H. Oh E. Sohn E. H. Lee A. Y. (2016). Treatment of Alzheimer's disease with repetitive transcranial magnetic stimulation combined with cognitive training: a prospective, randomized, double-blind, placebo-controlled study. J. Clin. Neurol. 12, 57–64. doi: 10.3988/jcn.2016.12.1.57, PMID: 26365021 PMC4712287

[ref27] Lee H. B. Lyketsos C. G. (2003). Depression in Alzheimer's disease: heterogeneity and related issues. Biol. Psychiatry 54, 353–362. doi: 10.1016/S0006-3223(03)00543-2, PMID: 12893110

[ref28] Li Y. Li X. Zhaung W. Yu C. Wei S. Li Y.. (2024). Relationship between cognitive function and brain activation in major depressive disorder patients with and without insomnia: a functional near-infrared spectroscopy (fNIRS) study. J. Psychiatr. Res. 169, 134–141. doi: 10.1016/j.jpsychires.2023.11.002, PMID: 38039687

[ref29] Li R. Zhang Y. Zhuo Z. Wang Y. Jia Z. Sun M.. (2021). Altered cerebral blood flow in Alzheimer's disease with depression. Front. Psychol. 12:687739. doi: 10.3389/fpsyt.2021.687739, PMID: 34305683 PMC8295555

[ref30] Lin Y. Jiang W. J. Shan P. Y. Lu M. Wang T. Li R. H.. (2019). The role of repetitive transcranial magnetic stimulation (rTMS) in the treatment of cognitive impairment in patients with Alzheimer's disease: a systematic review and meta-analysis. J. Neurol. Sci. 398, 184–191. doi: 10.1016/j.jns.2019.01.038, PMID: 30735817

[ref31] Mckhann G. Drachman D. Folstein M. Katzman R. Price D. Stadlan E. M. (1984). Clinical diagnosis of Alzheimer's disease: report of the NINCDS-ADRDA work group under the auspices of Department of Health and Human Services Task Force on Alzheimer's disease. Neurology 34, 939–944. doi: 10.1212/WNL.34.7.939, PMID: 6610841

[ref32] Metzger F. G. Schopp B. Haeussinger F. B. Dehnen K. Synofzik M. Fallgatter A. J.. (2016). Brain activation in frontotemporal and Alzheimer's dementia: a functional near-infrared spectroscopy study. Alzheimers Res. Ther. 8:56. doi: 10.1186/s13195-016-0224-8, PMID: 27931245 PMC5146884

[ref33] Mutz J. Vipulananthan V. Carter B. Hurlemann R. Fu C. H. Y. Young A. H. (2019). Comparative efficacy and acceptability of non-surgical brain stimulation for the acute treatment of major depressive episodes in adults: systematic review and network meta-analysis. BMJ 364:l1079. doi: 10.1136/bmj.l1079, PMID: 30917990 PMC6435996

[ref34] Niu H. J. Li X. Chen Y. J. Ma C. Zhang J. Y. Zhang Z. J. (2013). Reduced frontal activation during a working memory task in mild cognitive impairment: a non-invasive near-infrared spectroscopy study. CNS Neurosci. Ther. 19, 125–131. doi: 10.1111/cns.12046, PMID: 23279823 PMC6493442

[ref35] Noda T. Yoshida S. Matsuda T. Okamoto N. Sakamoto K. Koseki S.. (2012). Frontal and right temporal activations correlate negatively with depression severity during verbal fluency task: a multi-channel near-infrared spectroscopy study. J. Psychiatr. Res. 46, 905–912. doi: 10.1016/j.jpsychires.2012.04.001, PMID: 22572569

[ref36] Orgeta V. Tabet N. Nilforooshan R. Howard R. (2017). Efficacy of antidepressants for depression in Alzheimer's disease: systematic review and meta-analysis. J. Alzheimers Dis. 58, 725–733. doi: 10.3233/JAD-161247, PMID: 28505970 PMC5467718

[ref37] Padovani A. Antonini A. Barone P. Bellelli G. Fagiolini A. Ferini Strambi L.. (2023). Exploring depression in Alzheimer's disease: an Italian Delphi consensus on phenomenology, diagnosis, and management. Neurol. Sci. 44, 4323–4332. doi: 10.1007/s10072-023-06891-w, PMID: 37402937 PMC10641046

[ref38] Pinti P. Tachtsidis I. Hamilton A. Hirsch J. Aichelburg C. Gilbert S.. (2020). The present and future use of functional near-infrared spectroscopy (fNIRS) for cognitive neuroscience. Ann. N. Y. Acad. Sci. 1464, 5–29. doi: 10.1111/nyas.13948, PMID: 30085354 PMC6367070

[ref39] Quan W. Wu T. Li Z. Wang Y. Dong W. Lv B. (2015). Reduced prefrontal activation during a verbal fluency task in Chinese-speaking patients with schizophrenia as measured by near-infrared spectroscopy. Prog. Neuro-Psychopharmacol. Biol. Psychiatry 58, 51–58. doi: 10.1016/j.pnpbp.2014.12.005, PMID: 25542372

[ref40] Sabbagh M. Sadowsky C. Tousi B. Agronin M. E. Alva G. Armon C.. (2020). Effects of a combined transcranial magnetic stimulation (TMS) and cognitive training intervention in patients with Alzheimer's disease. Alzheimers Dement. 16, 641–650. doi: 10.1016/j.jalz.2019.08.197, PMID: 31879235

[ref41] Strangman G. Boas D. A. Sutton J. P. (2002). Non-invasive neuroimaging using near-infrared light. Biol. Psychiatry 52, 679–693. doi: 10.1016/S0006-3223(02)01550-0, PMID: 12372658

[ref42] Takemoto M. Ohta Y. Hishikawa N. Yamashita T. Nomura E. Tsunoda K.. (2020). The efficacy of sertraline, escitalopram, and Nicergoline in the treatment of depression and apathy in Alzheimer's disease: the Okayama depression and apathy project (ODAP). J. Alzheimers Dis. 76, 769–772. doi: 10.3233/JAD-200247, PMID: 32568205

[ref43] Teng E. Ringman J. M. Ross L. K. Mulnard R. A. Dick M. B. Bartzokis G.. (2008). Diagnosing depression in Alzheimer disease with the national institute of mental health provisional criteria. Am. J. Geriatr. Psychiatry 16, 469–477. doi: 10.1097/JGP.0b013e318165dbae, PMID: 18515691 PMC2989660

[ref44] Teselink J. Bawa K. K. Koo G. K. Sankhe K. Liu C. S. Rapoport M.. (2021). Efficacy of non-invasive brain stimulation on global cognition and neuropsychiatric symptoms in Alzheimer's disease and mild cognitive impairment: a meta-analysis and systematic review. Ageing Res. Rev. 72:101499. doi: 10.1016/j.arr.2021.101499, PMID: 34700007

[ref45] Trevizol A. P. Goldberger K. W. Mulsant B. H. Rajji T. K. Downar J. Daskalakis Z. J.. (2019). Unilateral and bilateral repetitive transcranial magnetic stimulation for treatment-resistant late-life depression. Int. J. Geriatr. Psychiatry 34, 822–827. doi: 10.1002/gps.5091, PMID: 30854751 PMC6488425

[ref46] Tsujii N. Mikawa W. Adachi T. Sakanaka S. Shirakawa O. (2021). Right prefrontal function and coping strategies in patients with remitted major depressive disorder. Prog. Neuro-Psychopharmacol. Biol. Psychiatry 108:110085. doi: 10.1016/j.pnpbp.2020.110085, PMID: 32889030

[ref47] Xiong S. Tu M. Wu X. Qu S. Chen N. Jin J.. (2023). Real-time hemodynamic changes in the prefrontal and bilateral temporal cortices during intradermal acupuncture for major depressive disorder: a prospective, single-center, controlled trial protocol. Neuropsychiatr. Dis. Treat. 19, 2627–2638. doi: 10.2147/NDT.S435617, PMID: 38059202 PMC10697084

[ref48] Xue H. Li Y. X. Xiao Y. S. Fan W. H. He H. X. (2024). Repetitive transcranial magnetic stimulation for Alzheimer's disease: an overview of systematic reviews and meta-analysis. Front. Aging Neurosci. 16:1383278. doi: 10.3389/fnagi.2024.1383278, PMID: 38572153 PMC10987751

[ref49] Yan Y. Tian M. Wang T. Wang X. Wang Y. Shi J. (2023). Transcranial magnetic stimulation effects on cognitive enhancement in mild cognitive impairment and Alzheimer's disease: a systematic review and meta-analysis. Front. Neurol. 14:1209205. doi: 10.3389/fneur.2023.1209205, PMID: 37528850 PMC10389278

[ref50] Yang Z. Zhou Y. (2023). The repetitive transcranial magnetic stimulation in Alzheimer's disease patients with behavioral and psychological symptoms of dementia: a case report. BMC Psychiatry 23:354. doi: 10.1186/s12888-023-04864-z, PMID: 37221495 PMC10207599

[ref51] Yap K. H. Ung W. C. Ebenezer E. G. M. Nordin N. Chin P. S. Sugathan S.. (2017). Visualizing Hyperactivation in neurodegeneration based on prefrontal oxygenation: a comparative study of mild Alzheimer's disease, mild cognitive impairment, and healthy controls. Front. Aging Neurosci. 9:287. doi: 10.3389/fnagi.2017.00287, PMID: 28919856 PMC5585736

[ref52] Zhang S. Liu L. Zhang L. Ma L. Wu H. He X.. (2022). Evaluating the treatment outcomes of repetitive transcranial magnetic stimulation in patients with moderate-to-severe Alzheimer's disease. Front. Aging Neurosci. 14:1070535. doi: 10.3389/fnagi.2022.1070535, PMID: 36688172 PMC9853407

[ref53] Zhou Y. (2019). Multiparametric imaging in neurodegenerative disease: Nova Medicine & Health. Available at: https://www.researchgate.net/publication/337199772

[ref54] Zhou Y. (2020). Imaging and Multiomic biomarker applications: Advances in early Alzheimer's disease: Nova Medicine & Health. Available at: https://www.researchgate.net/publication/349607788

